# Engineering controllable biofilms for biotechnological applications

**DOI:** 10.1111/1751-7915.13715

**Published:** 2020-11-29

**Authors:** Manisha Mukherjee, Bin Cao

**Affiliations:** ^1^ Singapore Centre for Environmental Life Sciences Engineering Nanyang Technological University Singapore 637551 Singapore; ^2^ School of Civil and Environmental Engineering Nanyang Technological University Singapore 639798 Singapore

Bacteria in natural and engineered habitats often live as multicellular aggregates embedded in a self‐produced matrix of extracellular polymeric substances (EPS), known as biofilms (Hall‐Stoodley *et al*., 2004; Flemming and Wuertz, 2019). Biofilms are central to several grand challenges that we need to address in the 21st century, for example, clean water access, as well as exert considerable economic impact on industry sectors ranging from environmental, agricultural to chemical, medical, energy and manufacturing. The term ‘biofilm engineering’ was first introduced in the early 1990s by the Center for Biofilm Engineering in Montana State University, where biofilm engineering broadly referred to fundamental and applied biofilm research driven by industrial, environmental and health issues. In 2019, the National Biofilm Innovation Centre (NBIC) of UK organized a biofilm engineering workshop, where the industrial and research community defined four key interventional strategies: Prevention, Detection, Management and Engineering, to tackle detrimental biofilms and utilize beneficial biofilms (https://www.biofilms.ac.uk/wp‐content/uploads/2019/11/NBIC‐Engineering‐Report‐Final.pdf). Among which, biofilm engineering refers to harnessing the beneficial uses of microbial communities by understanding the fundamentals of biofilm developmental process. This definition is more specific and narrows the focus of biofilm engineering down to the beneficial uses of biofilms.

Beneficial biofilms are mostly studied in the context of environmental biotechnology for purification of water and recovery of resources (Nicolella *et al*., 2000). The heterogeneity in structure and activity, and the lack of controllability of biofilm dynamics, limit the applications of beneficial biofilms. Effective control of biofilm structure and dynamics would enable and/or facilitate many biotechnological applications, for example efficient and controllable biofilm‐mediated biocatalysis for chemical production. Although several strategies have been reported in recent five years on engineering biofilms for improving biofilm‐mediated bioprocesses, biofilm engineering is still at the very early stage of the learning curve. Exploring new targets for engineering biofilms and applying new molecular tools for biofilm engineering will open up new avenues for facilitating practical applications of engineered biofilms. In this short article, we discuss targets and approaches that can be used to engineer controllable microbial biofilms.

## Targets for engineering biofilms

Biofilm development is controlled by complex regulatory networks that orchestrate different stages of the biofilm life cycle (Watnick and Kolter, 2000). Intercellular communications through quorum sensing (QS) signalling molecules play an important role in biofilm formation for many bacteria, where an intensified QS promotes the development of highly structured biofilms (Davies *et al*., 1998) while disrupting QS, or quorum quenching (QQ), reduced biofilm formation (Fetzner, 2015). Thus, QS serves as one target for controlling biofilm formation. For example, QS‐based biofilm engineering was deployed to control biofilm formation on water purification membranes. Wood *et al*. (2016) constructed a QS‐based gene circuit to engineer a self‐controllable, beneficial biofilm of *Escherichia coli* that limit its own thickness by sensing cell density via the LasI/LasR QS system of *Pseudomonas aeruginosa*. The engineered *E. coli* biofilm also produced nitric oxide that inhibited biofilm formation on membranes by other bacteria (Wood *et al*., 2016).

In addition to the intercellular QS systems, second messenger bis‐(3′‐5′)‐cyclic‐dimeric guanosine monophosphate (c‐di‐GMP) is also part of the regulatory networks orchestrating biofilm development. A decreased concentration of c‐di‐GMP reduces biofilm formation and a high level of c‐di‐GMP promotes the formation of biofilm (Hengge, 2009). Because c‐di‐GMP regulates biofilm formation and dispersal in a wide variety of bacteria, the implementation of c‐di‐GMP‐targeted biofilm engineering is expected to be compatible with diverse bacterial hosts. This strategy has been used in several studies to achieve enhanced performance of biofilm‐based bioprocesses. For example, Wu *et al*. (2015) engineered a *Comamonas testosteroni* biofilm through constitutively expressing YedQ, a c‐di‐GMP synthase from *E. coli*, and demonstrated enhanced biodegradation of organic pollutants by the engineered biofilm. In another study, Mukherjee *et al*. (2018) engineered an *E. coli* biofilm by introducing c‐di‐GMP‐based gene circuit to control biofouling of water purification membranes. The engineered biofilm controlled its own biofilm formation through the modulation of intracellular c‐di‐GMP concentration and inhibited biofouling/biofilm formation caused by other bacteria through the production of QQ enzyme (Mukherjee *et al*., 2018).

Another type of intracellular second messenger, cyclic AMP (cAMP), regulates metabolism and biofilm formation in several bacteria by activating cAMP receptor protein (CRP) (Ritzert *et al*., 2019). For instance, Kasai *et al*. (2019) overexpressed a cAMP synthase gene in *Shewanella oneidensis*, which greatly improves current generation by the electrochemically active biofilm in microbial fuel cells. Thus, by tuning the intracellular concentration of cAMP, the cAMP‐CRP regulatory system could be manipulated to achieve engineered biofilms with better performance. In addition to intracellular second messengers and QS signalling systems, small RNAs (sRNA) also play an important role in biofilm formation (Taylor *et al*., 2017). For example, a novel regulatory sRNA, SrbA, has been reported to upregulate biofilm formation by *P. aeruginosa*, while deletion of *srbA* reduced biofilm formation (Taylor *et al*., 2017), which provides a new target for biofilm engineering.

The hallmark of a biofilm is the self‐produced EPS matrix, which imparts numerous beneficial attributes to biofilms that are lacking in their planktonic counterparts (Flemming and Wingender, 2010). Matrix‐targeted biofilm engineering is another important route to harnessing the power of beneficial biofilms. Proteins present in the matrix serves as an excellent target for engineering. For example, Botyanszki *et al*. (2015) decorated the biofilm matrix with a catalytic enzyme, α‐amylase, using curli fibres of *E. coli* (Botyanszki *et al*., 2015). Immobilization of adsorptive or catalytic proteins on the biofilm matrix can be applied for biofilm‐mediated bioremediation, resource recovery, and biotransformation. Apart from proteins, extracellular polysaccharides and eDNA are also important components of the biofilm matrix and may serve as alternative targets for biofilm engineering (Flemming and Wingender, 2010). Advancement in our understanding of biofilm life cycle, diverse regulatory pathways, as well as biofilm matrix synthesis, structure, and function, would inform novel targets and approaches for engineering biofilms to improve biofilm bioprocesses.

## Exploring new tools for engineering biofilms

One of the major limitations that hampers the application of biofilm‐mediated bioprocesses lies in the uncontrollable dynamics of biofilm development, resulting in unpredictable and/or diminished performance (Hu *et al*., 2019). Controlling biofilm dynamics has been largely impeded due to inadequate knowledge of biofilm development and the unavailability of biological toolkits. Synthetic biology holds considerable promise for controlling biofilms by improving and expanding existing molecular tools, introducing novel functions to the system, modulating gene regulation and protein expression (Fu, 2006; Brenner *et al*., 2008). However, the shortage of well‐defined gene expression tools in a diverse range of bacteria restricts the scope of synthetic biology applications in potential bacteria relevant to biotechnological applications (Cao *et al*., 2019). Future efforts should focus on developing a systematic toolkit to expand our ability to genetically manipulate a diverse range of non‐model bacteria that have been shown to play important roles in biofilm‐mediated bioprocesses. For example, a recent study demonstrated that successful construction of genetic toolkit in a non‐model bacteria, *Chromobacterium violaceum*, facilitated the biorecovery of gold from electronic waste (Liow *et al*., 2020).

Synthetic gene circuits impart programmable functionalities in microorganisms that can be implemented in various biotechnological applications. However, an important factor influencing the performance of synthetic gene circuits is the interference from the host’s endogenous regulatory network. Therefore, it is desirable that the host itself should possess a low level of native signalling messengers/molecules to achieve an enhanced performance of the introduced gene circuit. Development of bacterial chassis by downregulating the gene expression of native signalling pathways, such as reduction of background c‐di‐GMP levels and QS signals, would improve the performance of gene circuits controlling biofilm development through mediating c‐di‐GMP and QS. CRISPR‐mediated gene silencing approach may offer an effective platform for systematically exploring the functions of genes involved in signalling pathways and facilitate genome‐scale manipulation to enhance the synthetic gene circuit activity for biofilm control.

Synthetic biology tools are widely used to design and control microbial communities by manipulating regulatory networks, regulating gene expression and engineering cell to cell interactions. By programming cellular behaviour of biofilms, synthetic biology approaches may enable broad applications ranging from understanding biofilm biology to engineering controllable biofilms for biocatalysis, energy generation and bioremediation. To achieve complex synthetic systems with high programmability and controllability, programmed gene circuits could be fine‐tuned by altering various input signals to generate desirable output/performance. Chemical inducers are often used to control the expression of programmed gene circuits; however, the potential toxicity, irreversibility and lack of spatial control limit their applications (Liu *et al*., 2018). In biocatalysis for chemical synthesis, adding chemical inducers would complicate downstream purification of chemical products. Recent advances in synthetic biology tools have harnessed the power of light to control bioactivities. Compared with chemical inducers, optogenetic tools are more desirable for controlling cellular behaviours as light is non‐invasive, easily controllable and cost‐efficient (Fenno *et al*., 2011). Optogenetic tools provide a promising strategy to precisely control biofilm dynamics by placing biofilm‐relevant genes under the control of light‐responsive switches (Mukherjee *et al*., 2018). Further research on programming bacterial cells to be more sensitive to lights and exploring other non‐chemical input signals, such as electrochemical and/or magnetic signals, is highly desirable for precise control of engineered biofilms for diverse biotechnological applications.

Another approach to engineering biofilms is to alter the activities of key regulatory proteins and/or enzymes that control biofilm development. In a previous study, an evolved variant of SdiA protein was generated through random mutagenesis and the variant led to higher biofilm formation in the presence of QS signals (Lee *et al*., 2009). Directed evolution is a powerful tool for protein engineering through mutation and selection, presenting another strategy to produce proteins with improved or novel functions (Kan and Joshi, 2019). With the advancement of molecular biology techniques, researchers can harness directed evolution in a targeted manner by applying mutations to specific proteins and selecting variants with enhanced functions. The improvement of synthetic gene circuit activity through directed evolution may serve as a powerful tool to engineer biofilms for enhancing biofilm‐mediated processes. In addition, it is important to build mathematical models combining genetic regulation and biofilm dynamics to guide efficient circuit designs and eventually predict the performance of engineered biofilms.

## Engineering multispecies biofilms

Biofilms in natural and engineered environments are generally composed of highly diverse microbial communities (Tan *et al*., 2017). Biofilms comprising of multiple species are more durable and resilient towards environmental stresses compared to single‐species biofilms (Lee *et al*., 2014; Tan *et al*., 2017). Furthermore, multispecies biofilms are known to facilitate complex biofilm‐mediated bioprocesses, comprising of multiple pathways, more efficiently than single‐species biofilms (Brenner *et al*., 2008). However, engineering multispecies biofilm communities to perform more complex and challenging biofilm‐mediated bioprocesses is at its infancy. The maintenance of long‐term robustness and stable interactions among community members is necessary to facilitate the deployment of engineered multispecies biofilms in complex bioprocesses (Johns *et al*., 2016). The spatial arrangement of different species within multispecies biofilm and cell‐to‐cell interactions plays a major role in building a stable community (Kim *et al*., 2008; Johns *et al*., 2016; Gordon *et al*., 2019). Spatial distribution of different microorganisms within a multispecies biofilm promotes population survival by producing public goods, facilitating interactions between different species and enhancing community resilience to environmental perturbations (Lee *et al*., 2014; Johns *et al*., 2016). The close spatial assortment of different bacterial species is advantageous to the community as a whole, for instance, degradation of recalcitrant organic molecules by mixed species biofilms were more efficient when the interacting species were spatially in close proximity (Wimpenny *et al*., 2000). A better understanding of the dynamics and localization of different species in structured biofilm communities will provide insights to engineering spatially defined controllable biofilm communities. As such, advances in microscopic techniques, microfluidic devices and 3D‐printing technologies will greatly improve our ability to study and design complex biofilm communities with spatially defined patterns.

Division of labour across different individuals in a consortium enhances a specialized function by reducing the metabolic burden of each population (Tsoi *et al*., 2018). Several studies have reported that by compartmentalizing the task among the microbial partners through mutualistic interactions, thereby reducing the number of steps required to achieve the same outcome (Minty *et al*., 2013; Wang *et al*., 2015). Beyond metabolism, QS system is also known to coordinate population‐level interactions (Abisado *et al*., 2018) and it can serve as an excellent target for regulating microbial interactions and provide useful components to construct synthetic networks for engineering multispecies biofilms (Davis *et al*., 2015; Johns *et al*., 2016). Nevertheless, engineered populations in multispecies biofilms may lose their functions over time, due to changing conditions, interaction with other species and genome evolution, which might ultimately decrease biofilm‐level performance. To reduce evolutionary decay of genetic circuits, strategies to avoid mutation‐prone designs and host mutation rates should be considered (Renda *et al*., 2014). Additionally, omics tools like metatranscriptomics will improve our understanding of the interactions within microbial communities and provide insights to engineering multispecies biofilms with stable microbial interactions. Furthermore, mathematical modelling of microbial interactions in biofilms is required to predict the spatiotemporal dynamics and performance of multispecies biofilms.
